# Cavitation-vibration coupling mechanism in ultrasonic guidewire vascular ablation

**DOI:** 10.1016/j.ultsonch.2025.107474

**Published:** 2025-07-24

**Authors:** Guang Yao, Maozhong Wu, Jianhua Lai, Youcheng Lv, Lijuan Zheng, Chengyong Wang

**Affiliations:** Guangdong Provincial Key Laboratory of Minimally Invasive Surgical Instruments and Manufacturing Technology, Guangdong University of Technology, Guangzhou 510006, China

**Keywords:** Vascular occlusion, Ultrasonic guidewire, Cavitation, Mechanical vibration, Tissue-specific ablation

## Abstract

Effective treatment of diverse vascular occlusions requires precise energy delivery and tissue-specific ablation strategies. This study systematically investigates the coupled mechanical vibration and cavitation mechanisms of a novel flexible ultrasonic guidewire during ablation of calcified, lipid-rich, and thrombotic occlusion mimics. Integrating numerical simulations and experimental validation, this work elucidates the dynamic interplay between ultrasonic parameters and tissue-specific ablation outcomes. For calcified mimics, mechanical vibrational impact is the dominant ablation mechanism, achieving substantial material removal primarily through fracture. Lipid-rich tissue ablation is driven by emulsification via cavitation microjets and acoustic streaming, generating microparticles with sizes of 10–250 μm, controllable by ultrasonic power. Thrombus ablation involves initial penetration followed by erythrocyte lysis, primarily mediated by transient cavitation. Crucially, guidewire bending significantly attenuates tip vibration amplitude, resulting in a reduction of 14.3–30.9 %, with titanium alloy exhibiting superior energy transmission stability under curvature compared to nickel-titanium. These findings highlight distinct, tissue-dependent ablation paradigms: mechanical fragmentation for hard tissues compared to cavitation and streaming induced emulsification or lysis for soft tissues. This mechanistic understanding is foundational for designing adaptive ultrasonic guidewires capable of adjusting energy delivery modes based on real time feedback of tissue characteristics, thereby enhancing the precision and efficacy of endovascular interventions.

## 1 Introduction

Vascular occlusion, primarily resulting from atherosclerosis and deep vein thrombosis (DVT), remains a leading cause of cardiovascular mortality worldwide [[1], [2], [3]]. Current therapeutic strategies, including pharmacological thrombolysis, open surgery, and conventional endovascular interventions, are limited by significant drawbacks. These include systemic bleeding risks associated with drug therapies, the invasiveness of surgical procedures [4], and complications such as distal embolization or vessel perforation related to current devices [5]. As a result, there is an urgent need for minimally invasive, targeted, and safer treatment options. In this regard, catheter-based ultrasound ablation has emerged as a promising technology. This technique utilizes high-frequency mechanical vibrations to fragment obstructive lesions (e.g., thrombi and atherosclerotic plaques), thereby restoring blood flow while minimizing thermal damage to surrounding vascular structures [6,7]. Its minimally invasive nature offers advantages for patients at high surgical risk, the elderly, and those unresponsive to thrombolytic therapies [8].

Previous studies have highlighted the potential of ultrasound in the treatment of vascular occlusions. Research on thrombolysis has shown that low-frequency (typically 20–40 kHz), high-intensity ultrasound can effectively dissolve both arterial and venous thrombi [[9], [10], [11], [12], [13]]. For example, ultrasonic guidewires have successfully achieved vascular recanalization with minimal reported vessel injury [11,12]. Additionally, techniques such as vortex ultrasound have been explored to enhance dissolution rates through shear stress mechanisms [14]. In a similar vein, studies targeting atherosclerotic plaques have demonstrated ultrasound’s ability to fracture calcified lesions and improve arterial compliance [[15], [16], [17], [18]]. Notably, ultrasound exhibits a degree of selectivity, with normal arterial tissue displaying greater resistance to ultrasonic damage than plaque [17], and successful fragmentation of calcified samples has been achieved with negligible thermal effects [19]. Moreover, advanced guidewire designs incorporating lateral vibrations have been investigated to expand the effective ablation zone [20,21].

Although these pioneering studies have collectively demonstrated the feasibility of ultrasound ablation, a comprehensive understanding of the distinct interaction mechanisms between ultrasound and various tissue types remains limited. This gap hinders the development of truly tissue-adaptive ultrasonic guidewire systems.

The therapeutic efficacy of ultrasound ablation arises from the synergistic effects of direct mechanical forces, cavitation (the formation and collapse of microbubbles), and acoustic microstreaming [[22], [23], [24], [25]]. Direct mechanical forces primarily affect brittle structures such as calcified plaques, leading to fragmentation through vibrational impact [26,27]. Cavitation contributes to thrombus disruption by breaking down the fibrin matrix via microjets and shear stress, and is particularly effective at lower ultrasound frequencies [28]. Simultaneously, acoustic microstreaming promotes tissue emulsification by inducing localized fluid motion [29]. The relative contributions of these mechanisms depend on both the ultrasound parameters and the physical properties of the target tissue [[30], [31], [32]]. For example, low-frequency ultrasound is generally more effective for thrombolysis due to enhanced cavitation activity, whereas high-amplitude, high-intensity ultrasound is more suitable for disrupting calcified plaques owing to its stronger mechanical effects [28,33].

Despite the considerable potential of ultrasonic vascular ablation, its clinical application faces significant challenges. The efficacy of this technology primarily relies on the complex cavitation-vibration coupling mechanism between the distal tip of the ultrasonic guidewire and the surrounding tissue, as well as the efficient delivery of energy to the distal end of the guidewire. However, this process is highly sensitive to in vivo factors, such as vascular tortuosity, which can lead to guidewire bending and consequently alter energy transfer efficiency. These variations in energy delivery may result in inadequate ablation or unintended damage to healthy tissue. Furthermore, a comprehensive understanding of how the cavitation-vibration coupling mechanism exerts differential effects on tissues with varying compositions and mechanical properties, as well as its response to changes in acoustic parameters, remains insufficiently explored [[34], [35], [36]].

This study systematically investigates the physical phenomena—cavitation, microjet formation, and vibrational impact—at the interface between the tip of an ultrasonic guidewire and various types of intravascular occlusions, including calcified plaques, lipid-rich plaques, and thrombi. Extending beyond previous research, this work elucidates how key ultrasonic parameters influence cavitation dynamics, jet formation, and vibration characteristics of the guidewire tip. Importantly, it distinguishes the dominant and secondary ablation mechanisms associated with different occlusion types based on their distinct physical properties. Based on these insights, an optimized energy control strategy for the ultrasonic guidewire tip is proposed. The paper is organized as follows: Section 2 describes the materials and methods, including guidewire fabrication, simulation of tip-induced cavitation and jetting, preparation of occlusion samples, and the experimental setup for ablation analysis. Section 3 presents the results, including characterization of guidewire vibrational and cavitation phenomena (Section 3.1), evaluation of ablation performance on thrombi, adipose tissue (representing lipid-rich plaques), and gypsum models (representing calcified plaques) (Section 3.2), and analysis the effects of guidewire bending and material properties on vibrational energy transmission, with implications for clinical optimization strategies (Section 3.3). Finally, Section 4 summarizes the key findings and contributions of this study.

## 2 Materials and methods

### 2.1 Development of ultrasonic guidewire

**Fig. 1:**
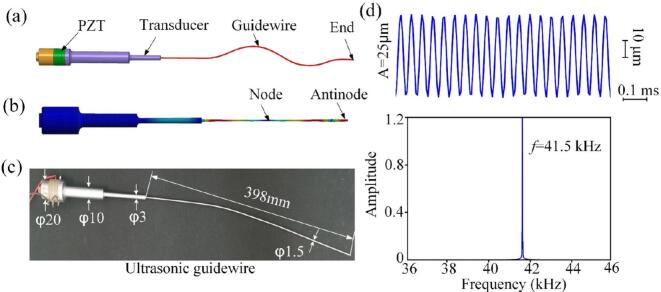
Development of the ultrasonic ablation instrument. (a) Schematic illustration of the ultrasonic guidewire. (b) FEA-based predictions for the longitudinal modal frequencies. (c) Geometrical dimensions of the ultrasonic guidewire. (d)Vibration amplitude at the guidewire end and the corresponding spectrum diagram.

The ultrasonic guidewire assembly consists of a sandwich-type transducer connected to a flexible guidewire (Fig. 1(a)). The transducer comprises a cylindrical back mass, a stack of piezoelectric ceramic rings (PZT), and a front mass that functions as an acoustic horn. Longitudinal ultrasonic vibrations are generated by applying a sinusoidal voltage across the PZT stack, exploiting the piezoelectric effect to convert electrical energy into high-frequency mechanical displacement. These vibrations are subsequently amplified by the horn and transmitted to the guidewire, which is attached to the distal end.

The driving system features an auto-tuning function, which performs a frequency sweep to identify and lock onto the resonant frequency of the system. The attachment of the guidewire has a significant impact on the overall system resonance and vibrational characteristics. The resulting vibration of the flexible guidewire is a combination of longitudinal and flexural modes.

A primary design objective was to maintain a stable longitudinal vibration frequency while enabling precise control of the flexural vibration frequency by adjusting the aspect ratio (length-to-diameter ratio) of the guidewire. Based on the one-dimensional longitudinal wave equation, the axial vibration displacement of each component within the transducer can be calculated as follows:

(1) $$\xi \left(x\right)=\left(A\sin\left(kx\right)+B\cos\left(kx\right)\right)/\sqrt{S}$$

Where *k* is the wave number of the ultrasonic wave, *S* is the cross-sectional area of the guidewire.

According to the beam flexural theory, the flexural vibration displacement of various components of the transducer can be derived as follows:

(2) y=Ach(mx)+Bsh(mx)+Csin(mx)+Dcos(mx)

Where $m=\sqrt{\omega /(c_{0}r)}$, *c*_0_ is the speed of sound wave, *r* is the radius of gyration, *ω* = 2π*f*, *f* is the frequency.

Based on Eqs. (1), (2), the structural parameters of the flexible ultrasonic guidewire have been preliminarily determined, with the detailed results presented in Table 1.

**Table 1:** Structural parameters for flexible ultrasonic guidewire.

Back mass	PZT	Front mass	Guidewire
20 mm	4 mm × 4	120 mm	398 mm × φ1.5 mm

To simulate the propagation of longitudinal ultrasonic vibrations in the flexible guidewire and optimize its design, Finite Element Analysis (FEA) was performed using Ansys Workbench, with axisymmetric models employed to improve computational efficiency. PZT-4 piezoelectric elements, polarized in the thickness direction (d_33_ mode), were used for modeling. Guidewires made from Titanium alloy (Ti-6Al-4 V) and Nickel-Titanium alloy (Niti), with diameters of 1.0 mm and 1.5 mm, were simulated. A fine mesh with a resolution of 0.02 mm was applied to both the transducer and guidewire models to accurately capture higher-order harmonic modes. The acoustic properties of Ti alloy and Niti are provided in Table 2. The simulation results revealed a dominant longitudinal vibration mode at 42 Hz, with the maximum amplitude at the guidewire tip, consistent with the design specifications.

**Table 2:** Acoustic properties of Ti Alloy and Niti.

Material	Density (kg/m^3^)	Sound velocity (m/s)	Impedance(m^2^·s)
Ti Niti	4430 8200	6176 5940	27.32 × 10^6^ 48.6 × 10^6^

Fig. 1(c) shows the assembled prototype of the flexible ultrasonic guidewire. Titanium alloy and Nickel-Titanium alloy (Niti) are preferred materials for manufacturing long, thin ultrasonic guidewires in biomedical applications due to their excellent biocompatibility. The flexible guidewire is mechanically coupled to the distal end of the horn via a heat-shrink interference fit.

The vibration displacement at the guidewire tip was measured using a Laser Doppler Vibrometer (LDV, Model PSV500, Polytec), which provides a minimum velocity resolution of 0.02 µm/s and a sampling period of 5 µs. The measured vibration displacement waveform at the guidewire tip, along with the corresponding frequency spectrum shown in Fig. 1(d), confirms the operational frequency and amplitude characteristics of the guidewire.

Ultrasound field measurements were conducted using a calibrated fiber-optic hydrophone (FOH, Precision Acoustics Ltd, Dorchester, U.K.) in a 3D scanning tank (UMS3, Precision Acoustics Ltd, Dorchester, U.K.), with an oscilloscope (TDS2024C, Tektronix, USA) used to acquire and display the time-varying signal. Acoustic pressure was measured at a location near the focal region in water. As the ultrasonic power increases, the pressure at the guidewire tip correspondingly rises, potentially reaching up to 2 MPa.

### 2.2 Simulation of tip-induced cavitation and microjets

To investigate the cavitation and acoustic jetting phenomena induced by an ultrasonic guidewire in liquid water (*ρ* = 1050 kg/m^3^), the dynamic behavior of a single cavitation bubble and the corresponding numerical models were developed using COMSOL Multiphysics. The collapse behavior of a single cavitation bubble near the tissue surface was simulated, with the initial bubble state in water. Following the studies by Li (2023) [37] and Han (2021) [38], the interface between the bubble and the tissue was modeled as an elastic boundary. A two-dimensional axisymmetric computational domain, measuring 8 × 8 mm, was used to represent the water medium. Environmental conditions were set to a pressure of 1.0133 × 10^5^ Pa and an initial temperature of 25 °C. A guidewire with a diameter of 1.5 mm was immersed to a depth of 3 mm within the liquid domain.

**Fig. 2:**
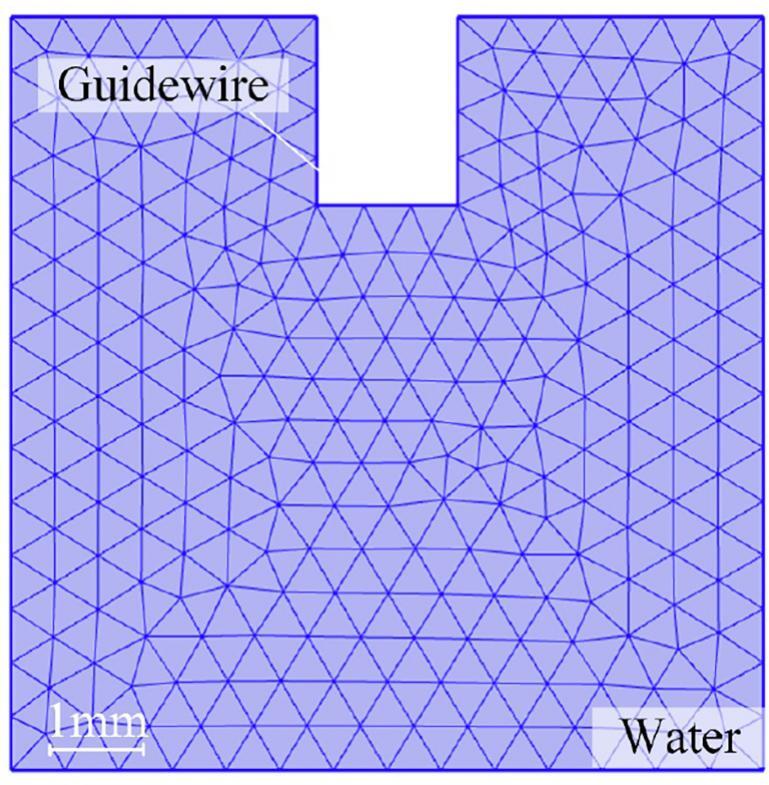
The guidewire acoustic field model and the details of the finite element mesh.

The axisymmetric approximation was adopted based on the cylindrical symmetry of the guidewire geometry and its uniaxial longitudinal vibration characteristics. However, this assumption may somewhat limit the accurate representation of complex flow behaviors in curved and non-axisymmetric regions. The computational domain was discretized using a free quadrilateral mesh, with element sizes ranging from 0.048 mm to 0.7 mm, to adequately resolve both the acoustic and flow fields (Fig. 2). Mesh independence was verified through simulations with varying mesh densities, which showed that mesh density had no significant effect on the results.

Vibrational boundary conditions were applied to the distal tip of the guidewire, with oscillation amplitudes set at 42 kHz frequency (∼5 μm, 10 μm, 15 μm, 20 μm, and 25 μm). These boundary conditions were based on experimental data from previous studies. A fixed time step of 1 μs was used to balance computational efficiency and solution accuracy. Sensitivity analysis indicated that the results were not significantly affected by the choice of time-stepping parameters. All other boundaries of the fluid domain were treated as rigid boundaries to simulate total acoustic reflection.

To understand the formation of cavitation clouds near the ultrasound guidewire tip, it is crucial to recognize that cavitation is driven by a multi-bubble cloud, rather than a single bubble. In this study, high-speed camera recordings were used to capture and observe the cavitation cloud phenomenon. The behavior of multi-bubble cavitation clouds is inherently complex, involving interactions both between the bubbles themselves and between the bubbles and the surrounding tissue. This paper focuses on the bubble-tissue interaction, simplifying the analysis to single-bubble dynamics through the Rayleigh-Plesset equation. Although this simplification limits the model, it provides valuable insights into localized effects, such as mechanical stress and shock waves. However, while single-bubble simulations offer useful data, they cannot fully account for the interactions between multiple bubbles, which significantly influence the overall dynamics of the cavitation cloud.

In addition, numerical simulations of cavitation bubble dynamics were performed using MATLAB to investigate the impact of key parameters on bubble behavior. These simulations were based on the Rayleigh-Plesset equation and other related models, with solutions obtained through the Runge-Kutta method [39]. The temporal integration of the governing ordinary differential equation(s), such as the Rayleigh-Plesset equation, was carried out using the Runge-Kutta numerical method. The simulations began at *t* = 0, with the acoustic driving pressure initiating from its negative phase, and the initial conditions set as a bubble at rest in equilibrium. The liquid was modeled as standard water at ambient temperature, with the following properties: specific heat ratio (*k*) = 1.33, density (*ρ*) = 998 kg/m^3^, surface tension (*S*) = 0.072 N/m, dynamic viscosity (*μ*) = 0.001 kg/(m·s), ambient pressure (*P_0_*) = 101325 Pa, and saturated vapor pressure (*P_v_*) = 2330 Pa.

### 2.3 Preparation of vascular occlusion samples

To simulate the various types of intravascular atherosclerotic plaques, three tissue-mimicking phantoms were fabricated—representing calcified, lipid-rich, and thrombotic regions—and their mechanical and acoustic properties were carefully characterized. A detailed description is provided below:

To replicate calcified lesions, a plaster-based phantom was created by mixing anhydrous calcium sulfate (CaSO_4_) powder with deionized water in a 10:3 wt ratio, forming a homogeneous paste. The mixture was then cast into cylindrical molds (6 mm in diameter, approximately 10 mm in length) and dried in an oven at 60 °C for 12 h. Nanoindentation analysis revealed an elastic modulus of 23–28 GPa and a microhardness of 573 ± 100 MPa for the phantom. These values align closely with the mechanical properties of human vascular calcifications, as reported by Ebenstein et al. (2009) [40]—an elastic modulus of 17.7–27.55 GPa and microhardness of 597 ± 158 MPa. As such, the phantom is considered an accurate mechanical representation of human calcified plaques. Furthermore, the acoustic velocity of the plaster is approximately 5800 m/s.

Fresh pig adipose tissue was obtained from a local slaughterhouse, cut into blocks approximately 6 × 6 × 10 mm^3^, to simulate lipid-rich plaques. Its elastic modulus is approximately 3.25 MPa [41], which is close to that of human adipose tissue (6.81 ± 3.83 MPa) [42], and the acoustic velocity is approximately 1450 m/s.

The thrombus model was made using whole cow blood. A 2.2 % CaCl_2_ solution was prepared by dissolving 2.2 g of anhydrous calcium chloride in 100 mL of physiological saline. The blood was mixed with the solution in a 4:1 ratio, stirred, and then poured into PVC tubes. It was left to stand at room temperature for 12 h and then refrigerated for 24 h to form the thrombus. After being cut into the desired size, the elastic modulus was measured to be between 11 and 42 kPa, consistent with reported values for human thrombus [43], and the acoustic velocity was approximately 1550 m/s.

### 2.4 Experimental setup for ultrasonic ablation observation

**Fig. 3:**
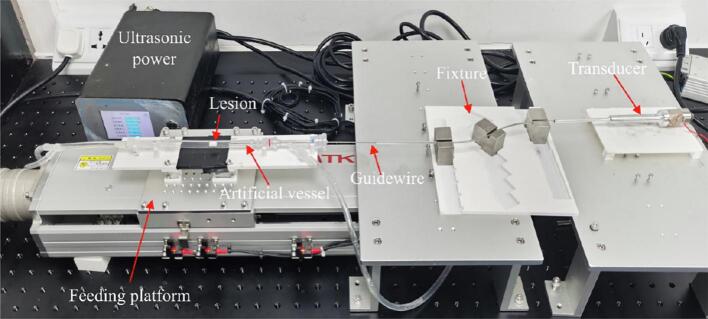
Experimental platform designed for observing ultrasonic guidewire ablation of vascular occlusion phantoms under controlled curvature and flow conditions.

To evaluate the mechanisms of ultrasound ablation on different types of obstruction models, we established an experimental platform that simulates a curved intravascular environment (Fig. 3). At the core of the setup is a transparent, flexible tube that mimics the vascular lumen. A medical three-way stopcock is integrated into the tubing circuit to serve as an access port. The ultrasound ablation device is securely mounted using an adjustable fixture. To replicate tortuous blood vessels, the system is designed to bend the guidewire along a curve with an 80 mm radius of curvature. A servo motor drives a lead screw mechanism to advance the ultrasound guidewire at a constant speed of 0.15 mm/s, ensuring consistent contact with the target model during ablation. A peristaltic pump circulates water through the system at a controlled flow rate to simulate physiological hemodynamics. To visualize the ablation process inside the transparent tube, a high-speed camera (MEMRECAM ACS-3, NAC, Japan) is positioned at a slight radial angle, capturing footage at 16,000 frames per second with a resolution of 1280 × 896 pixels, allowing for clear observation of the dynamic interactions between the guidewire tip and the model material.

### 2.5 Statistical analysis

Each experimental condition was performed five times to ensure statistical reliability. Statistical analyses, including analysis of variance (ANOVA), were conducted using GraphPad Prism 5.0 (GraphPad Software, USA). Data are expressed as the mean ± standard deviation (SD). One-way ANOVA was employed to compare multiple groups, with pairwise comparisons carried out using an independent samples *t*-test when appropriate. A p-value of less than 0.05 was considered statistically significant.

## 3 Results

### 3.1 Characterization of guidewire vibration and cavitation

#### 3.1.1 Cavitation effects of ultrasonic guidewire

Ultrasonic waves trigger the formation, expansion, contraction, and violent collapse of cavitation bubbles. As these bubbles expand several times their initial size, they eventually implode, generating shock waves and microjets that can cause significant damage to nearby solid surfaces. Soft tissues are highly susceptible to cavitation-induced damage [44].

The equilibrium radius of the bubble, along with the oscillation frequency and acoustic intensity of the sound field, influences whether cavitation is transient or steady, particularly in terms of the relationship between the sound field oscillation frequency (*ω*) and the bubble natural frequency (*ω_n_*). When (*ω*) is much smaller than (*ω_n_*), Blake’s static response criterion [45] indicates that once the external pressure change exceeds a threshold, a bubble with a steady-state radius (R) will undergo explosive growth, trigger transient cavitation and yield the Blake cavitation threshold (P_B_), as shown in Eq.3. However, when (*ω*) is close to (*ω_n_*), the dynamic variations in the sound field must be considered to determine whether the variation period is sufficient to cause unstable bubble growth. If the bubble undergoes transient cavitation, with its rupture speed approaching the speed of sound in the liquid, the bubble motion is treated as a transient event, with the maximum radius approximately 2.3 times the initial radius (R_m_ = 2.3R)) [46]. This serves as a criterion for identifying transient cavitation, which corresponds to the transient cavitation sound pressure threshold (P_T_).

The cavitation threshold of Blake is:

(3) $$P_{B}=P_{0}-P_{v}+\frac{4\sigma }{3\sqrt{3}R}{\left[1+\left(P_{0}-P_{v}\right)\frac{R}{2\sigma }\right]}^{-\frac{1}{2}}$$

Here, *R* is the instantaneous bubble radius, *σ* is the surface tension, P_0_ is the static liquid pressure, and P_v_ is the vapor pressure inside the bubble.

The transient cavitation threshold is:

(4) $$R_{T}=\left\{\begin{array}{c} \frac{0.13}{f}{\left(\frac{P_{0}}{\rho }\right)}^{1/2}\left[\frac{p-1}{\sqrt{p}}\left[1+\frac{2}{3}{\left(p-1\right)}^{1/3}\right]\right];p⩽11\\ \frac{0.3}{f}{\left(\frac{P_{0}}{\rho }\right)}^{1/2}\frac{2}{3}{\left(p-1\right)}^{1/2};p⩽11 \end{array}\right\}$$

Where p=P_T_/P_0_.

**Fig. 4:**
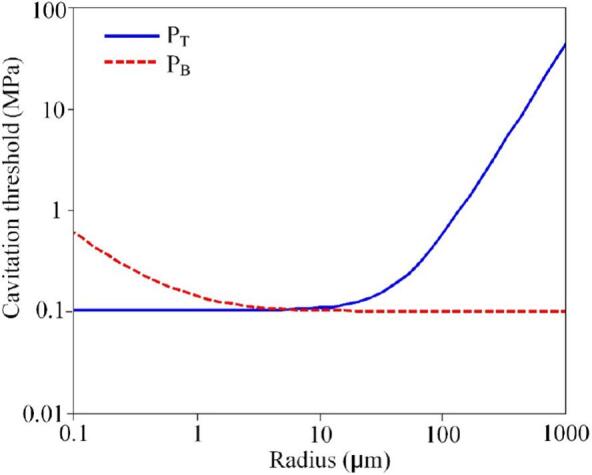
Curves showing the relationship between transient cavitation thresholds and equilibrium radius.

Based on Eqs. (3), (4), the relationship between the transient cavitation sound pressure threshold and the bubble equilibrium radius can be derived, as shown in Fig. 4. By considering the sound field generated by the ultrasonic guidewire, we can identify the type of cavitation induced and evaluate the likelihood of transient cavitation. For a guidewire frequency of 40 kHz, attention is given to bubbles that resonate at this frequency, particularly those with a natural frequency of 40 kHz. According to Eq. (4), the equilibrium radius of these resonant bubbles is calculated to be (R_n_ = 84.5 μm).

From the curve depicted in Fig. 4, the thresholds for transient cavitation can be extracted: P_T_ = 0.389 MPa. The acoustic pressure amplitude at the tip of the ultrasonic guidewire exceeds P_T_, indicating that the acoustic pressure generated by the guidewire is significantly higher than the pressure required to trigger transient cavitation. Therefore, it can be concluded that the ultrasonic guidewire can induce strong transient cavitation phenomena.

**Fig. 5:**
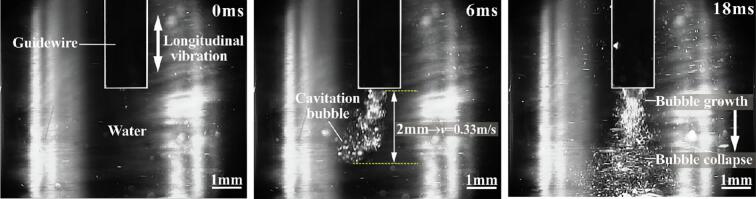
Cavitation bubble behavior at the guidewire tip.

To quantify the cavitation effect, we employed high-speed imaging to track bubble dynamics, including changes in bubble density over time. These measurements are essential for comparing transient and stable cavitation and for understanding their effects on tissue fragmentation. High-speed imaging technology enables real-time monitoring of bubble formation, growth, and collapse. As shown in Fig. 5, the guidewire tip generates many cavitation bubbles that rapidly grow and rupture at a distance from the tip, producing shockwaves and microjets that disrupt or ablate tissue. By measuring bubble density, we observed that at t = 6 ms, the bubble density induced by the guidewire tip was 2.1 %, and at t = 18 ms, the density had increased to 5.4 %.

**Fig. 6:**
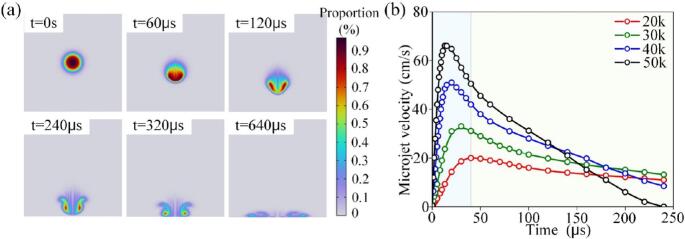
Collapse process of cavitation bubbles. (a) Cavitation bubble generation process. (b) Motion curves of cavitation bubbles under varying ultrasonic frequencies.

The growth and collapse process of a cavitation bubble, including the prominent formation of microjets during collapse, is illustrated in Fig. 6(a). Higher ultrasonic frequencies are associated with higher microjet velocities, as depicted in Fig. 6(b).

**Fig. 7:**
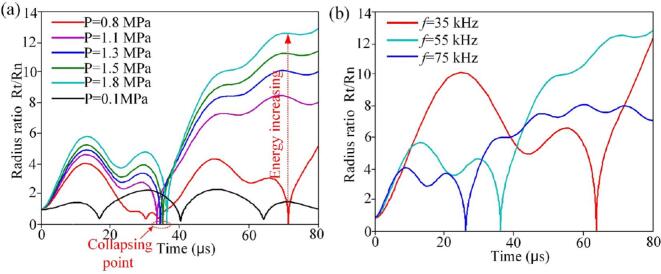
Collapse diameter of cavitation bubbles. (a) Motion curves of cavitation bubbles under various sound pressures. (b) Motion curves of cavitation bubbles at various ultrasound frequencies.

The interaction between the acoustic field and the bubble is influenced by both sound pressure and frequency. Simulations of bubble growth under various conditions show that increasing frequency reduces the bubble's radial amplitude and growth time, as illustrated in Fig. 7(a). At higher frequencies, the bubble's maximum size is smaller due to a shorter negative pressure phase, which limits growth. In contrast, lower frequencies promote greater bubble growth and enhance cavitation effects.

When a bubble collapses to a radius approaching zero (rupture point shown in Fig. 7(b)), the acoustic pressure further intensifies the bubble motion. The maximum radius can exceed 2.3 times the initial radius, confirming the presence of transient cavitation. At lower acoustic pressures, the collapse duration increases; when the pressure falls below the cavitation threshold, the phenomenon shifts from transient to stable cavitation. For example, at an acoustic pressure of P_A_ = 0.1 MPa, bubble collapse is minimal, indicating that a higher pressure is required to induce effective transient cavitation.

To achieve optimal cavitation effects, a combination of high acoustic pressure, low frequency, and resonance between the natural frequency of the bubble and the acoustic field is essential. When tissue properties are fixed, cavitation intensity can be enhanced by increasing the power output of the ultrasonic guidewire, lowering the operating frequency, or selecting a guidewire with a resonant frequency that matches the distribution of bubble sizes.

#### 3.1.2 Vibration impact of ultrasonic guidewire

The high-frequency vibrations generated by the ultrasonic guidewire tip exert force on the contact surface, causing pressure fluctuations within the surrounding medium. These fluctuations induce periodic perturbations in the medium particles, generating pressure waves that radiate outward and establish an acoustic field. Within this ultrasonic field, the liquid pressure corresponds to the acoustic pressure produced by the guidewire. The vibration displacement equation for the ultrasonic guidewire is given by [47]:

(5) S=Asinwt=Asin2πft

Where *S* is the displacement of the guidewire end, *A* is the ultrasound amplitude, and *f* is the ultrasound frequency. The vibration acceleration is then:

(6) $$a=S^{\prime \prime }=-4\pi^{2}f^{2}A\sin2\pi ft$$

The amplitude (*A*) at the ultrasonic guidewire tip was measured to be 10 μm, and the operating frequency (*f*) was 40 kHz. The guidewire tip exhibits localized circular vibration with a diameter of approximately 1.5 mm and can be modeled as a circular sound source. The mass of this vibrating region is estimated to be 3.5 × 10^−4^ kg. The maximum pressure exerted on the end face at the contact interface can be calculated as follows [48]:

(7) $$P=\mathrm{ma}/S=\left(3.53\times 10^{-4}\times 5.5\times 10^{5}\right)/\left(3.14\times 0.75\times 0.75\right)=11000N/cm^{2}$$

Assuming the density of human soft tissue is approximated by that of water (*ρ* = 1000 kg/m^3^) and the speed of sound in this medium is C = 1600 m/s, the sound intensity (I) can be derived as [49]:

(8) $$I=P^{2}/2\rho C=11000^{2}/\left(2\times 1000\times 1600\right)=37.8W/cm^{2}$$

The established cavitation threshold of acoustic intensity is 0.75 W/cm^2^ for human tissue fluid and 1 W/cm^2^ for plasma. The maximum acoustic intensity generated by the ultrasonic guidewire vibration significantly exceeds the cavitation threshold for most human tissues. The minimum sound pressure (P_min_) required to induce cavitation is:

(9) $$P_{\min}=\sqrt{I_{\min}\rho C}=154.92\text{kPa}$$

The near-field length (N) of a circular sound source is [50]:

(10) N=S/πλ=Sf/πC=13.2μm

The intensity within the near-field zone is relatively low. Therefore, to evaluate the potential for cavitation, the far-field sound pressure must be considered. The maximum distance (*r*_max_) at which cavitation can be induced is subsequently calculated by relating the far-field pressure decay to the cavitation threshold pressure:

(11) $$r_{\max}=\mathrm{PSf}/P_{\min}C=2.93mm$$

This calculated distance is relatively small. As a non-focused ultrasound surgical instrument, the limited maximum cavitation induction distance of the ultrasonic guidewire implies that the propagation and effective range of the sound waves are constrained.

**Fig. 8:**
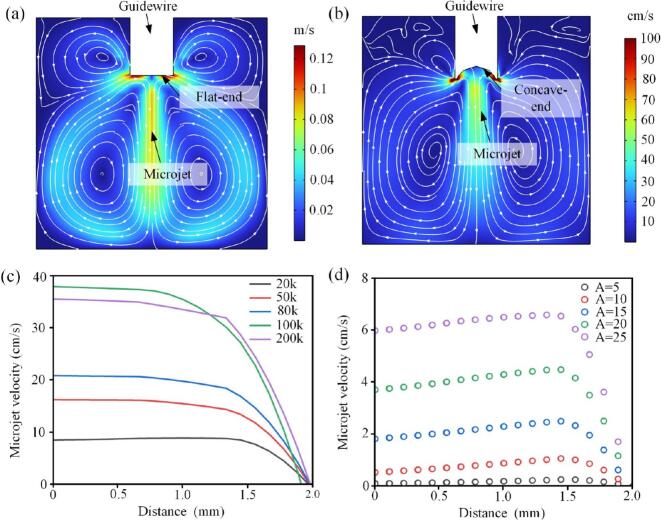
Acoustic streaming on the tip of ultrasonic guidewire. (a) Streaming produced by the flat-tip guidewire. (b) Streaming produced by the concave-tip guidewire. (c) Influence of ultrasonic frequency on streaming velocity. (d) Influence of ultrasonic amplitude on streaming velocity.

The high-frequency vibrations at the tip of the guidewire generate an acoustic flow, with the flow pattern influenced by the shape of the tip. A guidewire with a flat tip produces a jet-like acoustic flow that covers a wider distribution range (Fig. 8(a)), whereas a guidewire with a concave tip creates a more concentrated acoustic flow at the concave area, though with lower intensity (Fig. 8(b)).

Analysis of the acoustic flow velocity relative to distance for the flat-tip guidewire reveals that the flow velocity varies with frequency. Between 20 and 80 kHz, the flow velocity remains relatively stable within 1.5 mm from the tip. However, at 100 kHz, the stable region contracts to 1.0 mm, and the intensity diminishes. In general, ultrasound vibration energy increases with amplitude and frequency; however, the relationship between frequency and flow velocity is not linear. Below 100 kHz, the flow velocity increases with frequency, while above 100 kHz, the flow velocity decreases as the frequency rises (Fig. 8(c)).

The effect of vibration amplitude on flow velocity is linear. Within the 1.5 mm observation range, the flow velocity curves at different amplitudes are similar, suggesting that the effective range is largely unaffected by amplitude. However, the flow velocity is directly proportional to the vibration amplitude of the guidewire: the larger the amplitude, the higher the flow velocity (Fig. 8(d)).

### 3.2 Ultrasound ablation performance for different occlusions

#### 3.2.1 Ultrasonic ablation of calcified gypsum

**Fig. 9:**
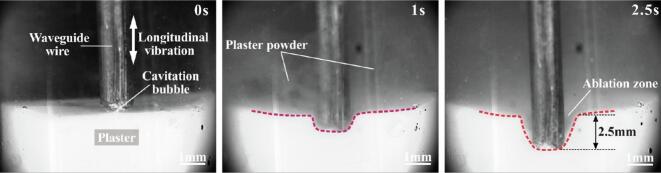
Ultrasonic ablation process of calcified gypsum.

At the start of the experiment (T = 0 s), before the guidewire contacted the gypsum model, ultrasound was activated, and cavitation and acoustic streaming were observed at the guidewire tip. At T = 1.3 s, the acoustic streaming impacted the gypsum surface, causing small debris to detach and the liquid to become turbid, with the debris emulsified by cavitation. At T = 2.0 s, as the guidewire contacted the gypsum, the combined effects of cavitation and high-frequency vibration increased the ablation rate, causing a significant dispersion of debris. At T = 2.8 s, as the guidewire penetrated deeper, a cavity formed, and the pressure gradient drew the debris-laden suspension into the cavity until it was filled. The guidewire continued to advance until it completely penetrated the gypsum (Fig. 9). This process demonstrates the precision of ultrasonic ablation in material removal.

**Fig. 10:**
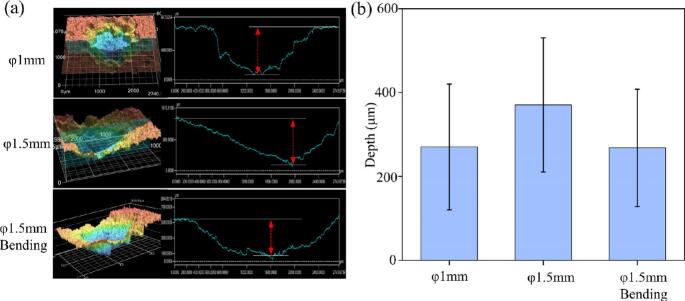
Ablation depth of gypsum. (a) Influence of guidewire diameter and bending on non-contact ablation depth in gypsum. (b) Hole diameter created by contact ablation with different guidewire diameters.

Non-contact ultrasonic ablation was performed on plaster using guidewires with diameters of φ1.5 mm and φ1.0 mm, resulting in the formation of regular pits on the ablated surfaces. The φ1.5 mm guidewire created pits with an depth of 370.78 ± 150 μm, while the φ1.0 mm guidewire produced pits with an average depth of 270.41 ± 120 μm (Fig. 10(a)), suggesting that the larger diameter guidewire offers greater ablation capability. Furthermore, a comparison of the ablation effects of the φ1.5 mm guidewire in both straight and bent configurations showed that ultrasonic energy remained effective even when the guidewire was bent, producing regular pits, though the depth was slightly reduced. These findings confirm that the ultrasonic guidewire can achieve effective ablation even under bending conditions.

Flexible guidewires (φ1.0 mm and φ1.5 mm) were used for contact ultrasonic ablation on a plaster model. The φ1.0 mm guidewire created a hole with a diameter of φ1.065 mm, which is 6.5 % larger than the guidewire diameter, while the φ1.5 mm guidewire produced a hole diameter of φ1.675 mm, 11.7 % larger (Fig. 10(b)). This enlargement is attributed to the flexural vibration component of the guidewire tip's motion. By adjusting the ultrasonic parameters, longitudinal vibration primarily facilitates penetration and obstruction clearance, while flexural vibration helps to expand the channel diameter, potentially improving the recanalization rate. This combined vibration mode enables effective blockage removal.

#### 3.2.2 Ultrasonic ablation of adipose tissue

**Fig. 11:**
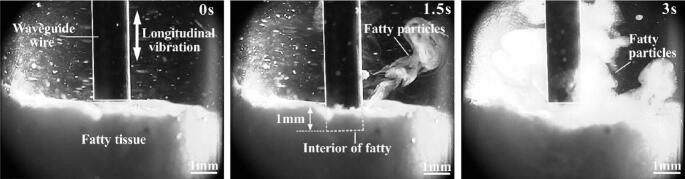
Ultrasonic ablation process of adipose tissue.

When animal fat was used to simulate lipid-rich obstruction, the ablation process was smoother. Initially, the liquid was clear, and cavitation occurred at the tip of the guidewire after ultrasonic activation. During the early stages, the fat showed minimal changes, with only small particles appearing in the liquid. As the guidewire neared the fat surface, high-frequency vibrations emulsified the fat, disrupting the surface layer and releasing particles. As the guidewire advanced, larger fat fragments were expelled, the number of emulsified particles increased, and the liquid became turbid. Eventually, the guidewire completely penetrated the fat obstruction (Fig. 11), demonstrating that ultrasound can effectively and stably ablate lipid-rich materials.

**Fig. 12:**
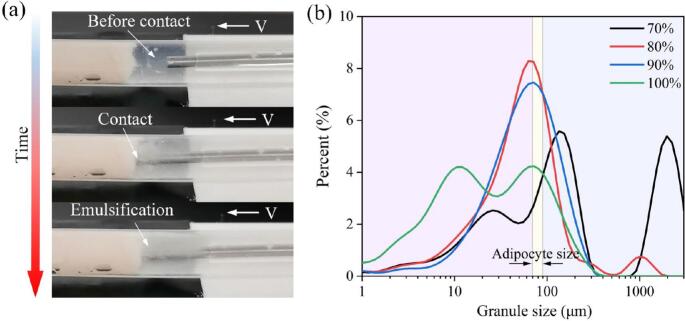
Ablation depth of adipose. (a) Ultrasonic ablation effect of adipose tissue. (b) Influence of ultrasonic power on particle size distribution.

Following the fat ablation experiment, collected particles were analyzed for size distribution (Fig. 12(a)). The adipose tissue was fragmented into particles ranging from 0.3 μm to 250 μm, with a primary concentration around 100 μm. This peak may relate to the approximate 70–90 μm diameter of the porcine abdominal adipocytes used. Most particles appeared to be intact cells liberated from the tissue matrix by the ultrasonic action. Ultrasonic power significantly influenced the resulting particle size distribution. Higher ultrasonic energy resulted in a broader size distribution with smaller particles predominating, while lower energy led to a more concentrated distribution with larger particles. At full power output, particles were concentrated in the 10–100 μm range. At lower power, the peak concentration shifted to sizes greater than 100 μm. Intermediate power output resulted in particles concentrated around 70–90 μm (Fig. 12(b)). Additionally, the ultrasonic action likely caused some cell lysis, contributing to the presence of smaller particles. These findings indicate that ultrasonic ablation parameters can influence the particle size distribution of ablated adipose tissue.

#### 3.2.3 Ultrasonic ablation of thrombi

**Fig. 13:**
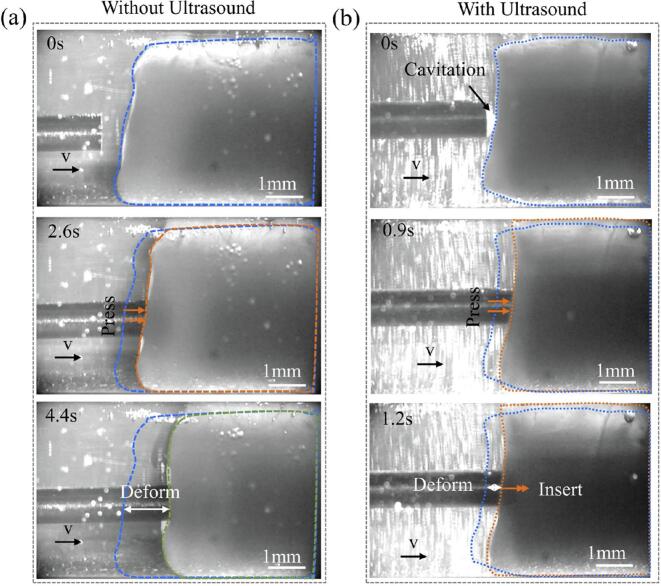
Comparison of guidewire interaction with thrombus. (a) Without ultrasound and (b) with ultrasound.

A comparison was made between guidewire interaction with thrombi with and without ultrasound activation. Without ultrasound (T = 0 s), the guidewire did not emit energy and left the thrombus undisturbed. Upon iNitial contact, the non-vibrating guidewire could not penetrate but instead caused thrombus compression. Continued advancement further compressed the thrombus without achieving penetration (Fig. 13(a)).

With ultrasound activated (T = 0 s), the guidewire emitted energy, causing slight thrombus deformation due to the acoustic field. As the guidewire advanced, it did not immediately penetrate the surface; its impingement and compression caused further deformation. At T = 1.180 s, the guidewire successfully penetrated the thrombus surface, accompanied by the release of red blood cells and a concurrent reduction in thrombus deformation (Fig. 13(b)). The guidewire then steadily ablated the thrombus from within until achieving complete penetration and recanalization. This highlights the critical role of ultrasonic energy output for effective thrombus penetration and ablation.

**Fig. 14:**
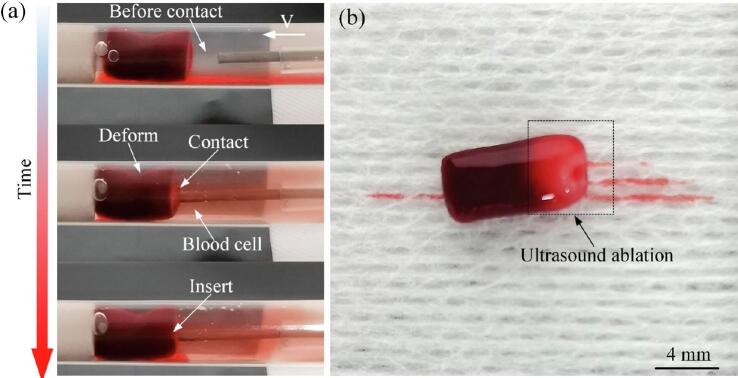
Ultrasonic ablation of thrombi. (a) Liquid turbidity changes. (b) Appearance of thrombus post-ablation.

Visually, the liquid was initially clear. As the guidewire approached and contacted the thrombus, high-frequency vibrations detached small amounts of blood, turning the liquid light red and slightly turbid. As the guidewire penetrated deeper, the ultrasonic ablation dissolved and destroyed cells within the thrombus, darkening the liquid to a deep red (Fig. 14(a)). When the thrombus was examined post-ablation, the treated area appeared lighter red and somewhat translucent due to the lysis of red blood cells and the creation of micro-channels by the ablation process (Fig. 14(b)). This demonstrates the lytic effect of ultrasonic ablation on thrombus structure.

The mechanism of ultrasonic ablation varies depending on the characteristics of the target obstruction. The entire ablation process is complex, involving a synergistic effect of mechanical vibration, cavitation, acoustic streaming, and potential ultrasonic thermal effects. The performance of the flexible intravascular ultrasonic guidewire developed in this study is influenced by key parameters, including the transducer design, guidewire material, diameter, and ultrasonic vibration characteristics (such as frequency, amplitude, and mode). These factors collectively determine the overall efficacy of ultrasonic ablation in treating the obstruction.

### 3.3 Tissue-specific dominant mechanisms

**Fig. 15:**
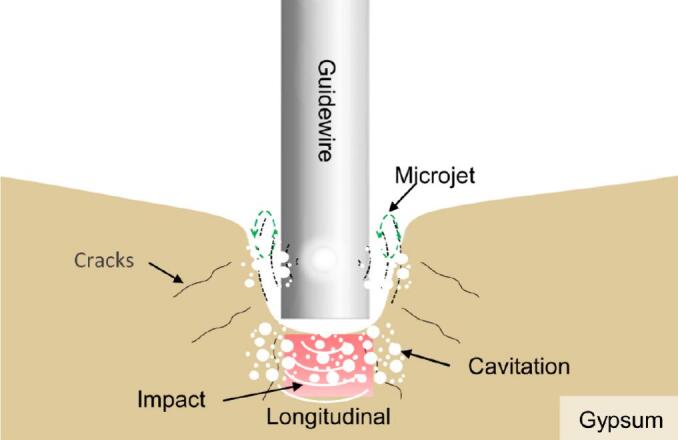
Schematic of ultrasonic ablation mechanism for calcified gypsum.

Ultrasonic ablation of the gypsum model, simulating calcified plaque, occurs in three distinct stages. In the pre-contact stage, prior to the guidewire contacting the model, acoustic streaming and cavitation effects already initiate material erosion near the model's surface. In the contact stage, once the guidewire tip contacts the gypsum, the liquid turbidity increases, and the ablation rate accelerates. This leads into the stable penetration stage, where the guidewire continues to advance, steadily removing material. Ultrasonic vibration proves especially effective on hard, brittle gypsum, primarily through the mechanical impact of the vibrating tip, which fractures and pulverizes the material. Concurrently, shear forces from acoustic streaming further promote erosion, while the high temperatures and pressures generated by cavitation crush larger fragments into finer particles, aiding in the emulsification of the ablated debris (Fig. 15).

**Fig. 16:**
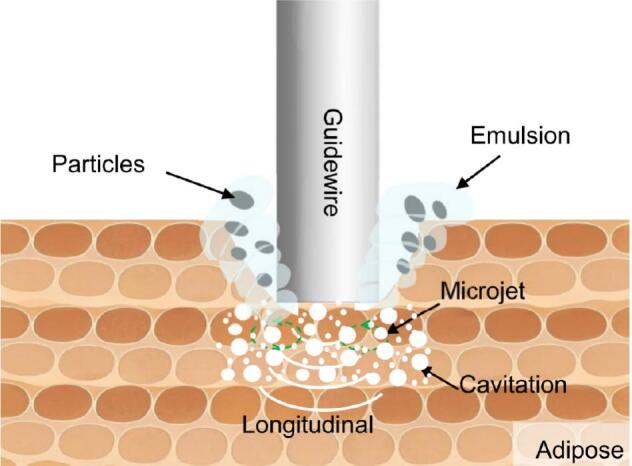
Schematic of ultrasonic ablation mechanism for adipose tissue.

In contrast to the ablation mechanism observed for hard, calcified gypsum, the direct mechanical impact of the ultrasonic vibration plays a less significant role in the removal of soft adipose tissue. Instead, acoustic streaming and cavitation effects emerge as the predominant mechanisms driving adipose tissue ablation. These phenomena facilitate the rapid emulsification of the adipose tissue, yielding an emulsion primarily composed of lipid droplets (Fig. 16).

**Fig. 17:**
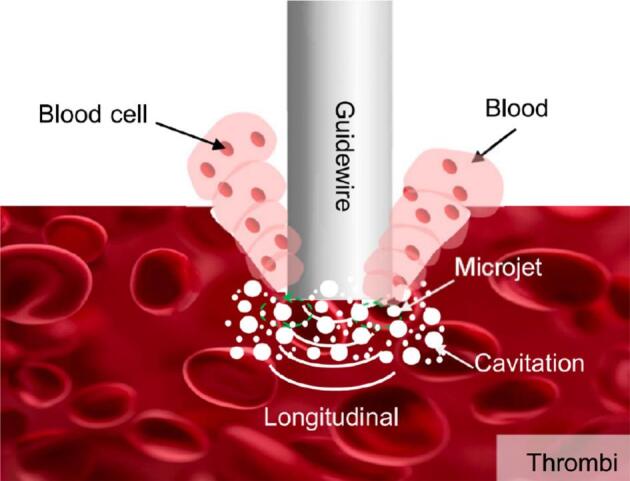
Schematic of ultrasonic ablation mechanism for thrombus.

The ultrasonic ablation of thrombus encompasses two primary phases. Initially, during the deformation phase, the advancing guidewire exerts pressure, causing significant deformation of the soft, elastic thrombus material prior to penetration. Following penetration, the process transitions to the internal ablation phase, where the thrombus partially recovers its shape around the guidewire, which then performs stable ablation within the thrombus core. Distinct from gypsum and adipose tissue, the soft and elastic nature of thrombus significantly influences the ablation mechanism. While the mechanical vibrational impact likely contributes to the initial penetration of the thrombus structure, its role in subsequent material removal is limited due to the tissue's elasticity; instead of efficient fragmentation, the impact primarily induces compression. Consequently, acoustic streaming and cavitation effects are identified as the principal mechanisms responsible for thrombus removal. However, unlike the emulsification of adipose tissue or the pulverization of gypsum, the dominant process here is lysis. This involves the breakdown of erythrocytes (red blood cells) within the clot matrix, leading to the release of intracellular fluid and cellular debris (Fig. 17).

### 3.4 Influence of guidewire design on tip vibration

**Fig. 18:**
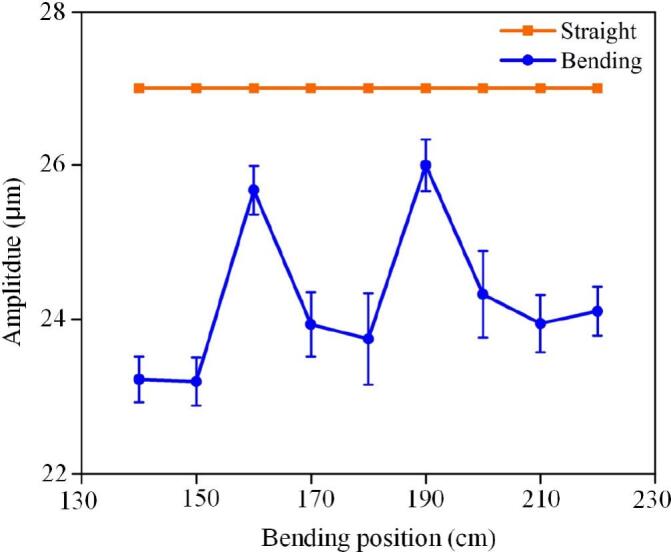
Amplitude at the end of guidewire under the bending and straight state. Error bars: ±SD.

The ultrasonic guidewire is a slender, flexible tool used for intravascular ablation, but it faces challenges when navigating long, tortuous blood vessels to reach the target lesion. The vibrational energy delivered to the distal tip, which is crucial for the efficiency of thrombus or plaque ablation, may be attenuated due to the guidewire length and the curvature of the vessel. The bending of the guidewire during operation significantly affects the tip amplitude, with the bending position being a key factor. Experiments (n = 5) show that moving the bending position along the guidewire leads to periodic changes in the tip amplitude. This is due to the interaction between the bending and the longitudinal ultrasonic standing wave within the guidewire: bending near the displacement node (where the amplitude is minimal) enhances the tip amplitude, while bending near the antinode (where the amplitude is maximal) diminishes it. The periodicity of this amplitude variation is approximately half the ultrasonic wavelength (λ/2) in the guidewire, highlighting the critical impact of the bending position relative to the standing wave distribution on energy transfer efficiency (Fig. 18).

**Fig. 19:**
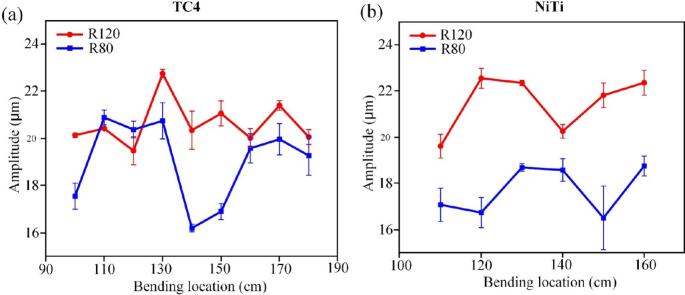
Influence of guidewire material on the end-tip amplitude under bending (n = 5). (a) Amplitude variation for the titanium guidewire as a function of bend position. (b) Amplitude variation for the nickel-titanium guidewire as a function of bend position. Error bars: ±SD.

The material properties of the guidewire play a crucial role in determining its ultrasonic transmission efficiency, particularly under bent conditions. Commonly used biocompatible materials, such as Nickel-Titanium (Niti) and Titanium (Ti) alloys, exhibit distinct ultrasonic performance characteristics. Experimental comparisons (n = 5) revealed that while bending induces tip amplitude fluctuations in both materials, the spatial period of these fluctuations is significantly shorter for Niti guidewires (approximately 40 mm) compared to Ti guidewires. This difference arises directly from the varying acoustic velocities (c) and the resultant ultrasonic wavelengths (λ = c/f) inherent to each material. Additionally, Niti guidewires exhibit substantially higher sensitivity to bending-induced attenuation. The maximum reduction in tip amplitude reached 30.9 % ± 2.1 % for Niti, which is considerably higher than the 14.3 % ± 1.8 % attenuation observed in Ti under similar conditions (Fig. 19). Statistical analysis confirmed a significant difference between the materials (p < 0.05). These findings suggest that the intrinsic properties of Niti, such as its lower elastic modulus and potentially different internal damping characteristics, make its ultrasonic energy transmission less adaptable and more prone to greater losses when bent, compared to Ti alloys.

## 4 Conclusions

This study provides a comprehensive evaluation of the operational characteristics of a novel ultrasonic guidewire, focusing on its vibration and cavitation dynamics, testing its ablation efficacy on a range of occlusive materials, including calcified plaques, adipose tissue, and thrombus mimics, and analyzing how key design parameters affect energy transmission efficiency. The findings demonstrate that the ablation performance of the ultrasonic guidewire is primarily governed by the synergistic effects of cavitation, mechanical vibration, and acoustic streaming, with the predominant mechanism varying based on the physical properties of the target tissue. Calcified plaques are mainly disrupted by mechanical forces, while softer tissues such as adipose tissue and thrombi are primarily ablated through cavitation and acoustic streaming. This underscores the significance of tissue-specific interactions in ultrasonic ablation.

Compared to existing literature, which typically focuses on the ablation of calcified plaques, this study broadens the scope to include a variety of vascular occlusive tissues, such as soft thrombi, calcified plaques, and mixed lesions. For the first time, our research demonstrates effective ultrasonic ablation across different tissue types, highlighting the multifunctionality of the approach and reinforcing the theoretical foundation for customizing ultrasound parameters to target specific tissues. This not only enhances the precision of the procedure but also improves its overall efficiency.

The structural design of the guidewire plays a crucial role in energy transmission efficiency. The guidewire’s length influences resonance and signal attenuation, while the degree and location of bending affect vibration amplitude transmission. Material selection is also critical for ensuring energy stability. Titanium alloy guidewires outperform nickel-titanium guidewires in terms of energy transmission stability under bending conditions.

The analysis reveals that energy loss due to bending is largely reversible, with the guidewire returning to its original energy transmission state once straightened. However, repeated bending and fatigue over time may impact long-term performance. Future studies should assess the guidewire’s stability under prolonged use, particularly in terms of energy efficiency and mechanical resilience.

Although the ultrasonic guidewire demonstrates promising ablation performance, safety remains a key consideration. Ultrasonic ablation is typically carried out at low temperatures, minimizing the risk of thermal injury. However, future studies should include in vivo temperature monitoring to assess thermal dose and potential tissue damage. The current design, which avoids sharp blades or high-speed rotating components, reduces the risk of mechanical injury to the vessel wall. However, the biological effects of cavitation, particularly its potential to damage the endothelium, warrant further investigation. Future research should focus on evaluating cavitation-induced endothelial injury to ensure clinical safety.

In conclusion, this study elucidates the complex, multi-modal, and tissue-dependent mechanisms underlying ultrasonic guidewire ablation. The findings provide valuable insights into the roles of cavitation, mechanical vibration, and acoustic streaming in achieving effective tissue disruption. Moreover, these results offer essential guidance for optimizing device design, improving energy transmission efficiency, and ensuring the safety of endovascular therapies.

## CRediT authorship contribution statement

**Guang Yao:** Writing – original draft, Methodology, Investigation, Conceptualization. **Maozhong Wu:** Formal analysis, Data curation. **Jianhua Lai:** Validation, Software, Methodology, Investigation. **Youcheng Lv:** Supervision. **Lijuan Zheng:** Supervision, Project administration. **Chengyong Wang:** Supervision.

## Declaration of competing interest

The authors declare that they have no known competing financial interests or personal relationships that could have appeared to influence the work reported in this paper.

## Data Availability

Data will be made available on request.
